# Role of ATP-Small Heat Shock Protein Interaction in Human Diseases

**DOI:** 10.3389/fmolb.2022.844826

**Published:** 2022-02-16

**Authors:** Sandip K. Nandi, Alok Kumar Panda, Ayon Chakraborty, Shivani Rathee, Ipsita Roy, Subhashree Barik, Saswati Soumya Mohapatra, Ashis Biswas

**Affiliations:** ^1^ School of Health Sciences, University of Petroleum and Energy Studies, Dehradun, India; ^2^ School of Applied Sciences, KIIT Deemed to be University, Bhubaneswar, India; ^3^ School of Basic Sciences, Indian Institute of Technology Bhubaneswar, Bhubaneswar, India

**Keywords:** sHSPs, ATP, cataract, cardiovascular diseases, tuberculosis, leprosy

## Abstract

Adenosine triphosphate (ATP) is an important fuel of life for humans and *Mycobacterium* species. Its potential role in modulating cellular functions and implications in systemic, pulmonary, and ocular diseases is well studied. Plasma ATP has been used as a diagnostic and prognostic biomarker owing to its close association with disease’s progression. Several stresses induce altered ATP generation, causing disorders and illnesses. Small heat shock proteins (sHSPs) are dynamic oligomers that are dominantly β-sheet in nature. Some important functions that they exhibit include preventing protein aggregation, enabling protein refolding, conferring thermotolerance to cells, and exhibiting anti-apoptotic functions. Expression and functions of sHSPs in humans are closely associated with several diseases like cataracts, cardiovascular diseases, renal diseases, cancer, etc. Additionally, there are some mycobacterial sHSPs like *Mycobacterium leprae* HSP18 and *Mycobacterium tuberculosis* HSP16.3, whose molecular chaperone functions are implicated in the growth and survival of pathogens in host species. As both ATP and sHSPs, remain closely associated with several human diseases and survival of bacterial pathogens in the host, therefore substantial research has been conducted to elucidate ATP-sHSP interaction. In this mini review, the impact of ATP on the structure and function of human and mycobacterial sHSPs is discussed. Additionally, how such interactions can influence the onset of several human diseases is also discussed.

## Introduction

ATP is termed as energy currency of cells owing to its high energy phosphate bonds. It is used by several enzymes and structural proteins to mediate cellular processes. Besides energy production, ATP plays a pivotal role in synthesis of several macromolecules which are essential for cell survival. It acts as a switch to regulate chemical reactions and send messages. Mitochondria plays a key role in ATP synthesis by regulating oxidative phosphorylation (Bulthuis et al., 2019). Nitric oxide also regulates ATP synthesis by inhibiting cytochrome oxidase (Zhao et al., 2009). Low ATP synthesis is reported to correlate with faster tumor growth and its high invasive behavior (Granchi and Minutolo 2012). Although energy related dysfunction is not usually correlated with common diseases, but evidence suggests existence of such links in some disorders. Muscle, brain, liver, heart, and kidney that are primary energy consuming organs in human are often affected by mitochondrial dysfunction, which is a common cause for lower ATP levels (Kishikawa et al., 2018). Recent studies have demonstrated strategies to elevate levels of ATP by xanthine oxidoreductase inhibitors (Hosoyamada et al., 2016; Johnson et al., 2019; Kamatani et al., 2019) to treat disorders with ATP deficiency, associated with brain, heart, skeletal muscle, etc. (Ansari-Ramandi et al., 2017; Singh et al., 2017; Bredemeier et al., 2018; El-Bassossy et al., 2018; Ferrando et al., 2018; Singh and Cleveland 2018). Altogether, ATP is an important molecule that regulates metabolic processes and is closely associated with human diseases.

Small heat shock proteins (sHSPs) are the most strongly induced molecular chaperones under stress (Liu et al., 2015). It constitutes a divergent group within the class of HSPs characterized by a conserved “α-crystallin domain” (ACD) (Basha et al., 2012). The molecular mass of sHSPs ranges between 12–43 kDa and it can assemble into large, dynamic oligomers upto 1 MDa (Sharma and Santhoshkumar 2009). sHSPs are molecular chaperones that prevent stress induced aggregation of partially denatured proteins (Horwitz 1992; Raju et al., 2011). Some of the best explored sHSPs are archeal sHSPs such as HSP16.5 from *Methanococcus jannaschii*, HSP26 from *Saccharomyces cerevisiae*, α-crystallin and HSP27 (mammalian sHSPs), plant sHSP (HSP16.9 from wheat) and mycobacterial sHSP (HSP16.3 from *Mycobacterium tuberculosis*) (Horwitz 1992; Kim et al., 1998; Haslbeck et al., 1999; van Montfort et al., 2001; Fu et al., 2005; Lelj-Garolla and Mauk 2006). Besides the aggregation prevention ability, they also exhibit refolding ability like large heat shock proteins but are ATP hydrolysis independent (Jakob et al., 1993; Biswas and Das 2004). sHSPs confer thermotolerance to cells *in vivo* (Lavoie et al., 1995; Muchowski and Clark 1998; Valdez et al., 2002). Besides, sHSPs are over-expressed, which protect organisms and substrate proteins from other stress conditions such as oxidative and nitrosative stress (Wang and Spector 1995; Garbe et al., 1999). sHSPs exhibits anti-apoptotic function. They are also used to develop DNA vaccines which help in prevention and cure of infectious disease such as tuberculosis (Shi et al., 2010). Therefore, it is quite rational that these sHSPs can be used therapeutically in prevention of protein aggregation, apoptosis, and diseases.

In rat models, intravenously injected α-crystallin protects the retinal ganglion cells from apoptosis and promoted axonal regeneration after optic nerve crush (Ying et al., 2008; Wang et al., 2012; Wu et al., 2014). The retinal degeneration in the early phase of the autoimmune disease uveoretinitis can be prevented by systematic administration of αA (Saraswathy et al., 2010; Rao et al., 2012). In diabetic retinopathy, delivery of αA into the eyes of the mice decreased the vascular leakage and pericyte apoptosis, which is useful to stop the early lesions in the eyes (Kim et al., 2012). Delivery of cell penetration peptide tagged to α-crystallin into the cells exhibits improved protection against oxidative stress in lens epithelial cells (Mueller et al., 2013; Christopher et al., 2014). Apart from this, peptides derived from the sHSP (α-crystallin), act as mini chaperone and inhibit epithelial cell apoptosis and prevent cataract in experimental rat models, which can be of immense therapeutic use (Nahomi et al., 2013). Therefore, from the above discussion, it is reasonable to propose that sHSPs like α-crystallin and its peptides can be utilized as therapeutic agents.

On the contrary, several reports are available in the literature which demonstrates the detrimental effect of the over-expression of sHSPs in many diseases. For example, the over-expression of αB-crystallin in breast tumors leads to a shorter lifetime of the patients (Moyano et al., 2006). Subsequently, a recent study has identified a small molecule inhibitor for αB-crystallin, which binds to the ACD domain of the protein and inhibits the tumor growth in human breast cancer xenografts in mice (Chen et al., 2014). Similarly, the over-expression of HSP27 in breast cancer cells confers resistance to anti-cancer agents like doxorubicin (Oesterreich et al., 1993). Subsequently, attempts have also been made to inhibit the over-expression of HSP27 by using anti-sense or nucleotide-based therapies (Arrigo et al., 2007; Jego et al., 2013).

Vaccination is often used as a preventive therapeutic against pathogenic diseases. For example, *Mycobacterium bovis* Bacillus Calmette–Guérin (BCG), a live attenuated strain of *Mycobacterium bovis* is widely used as a vaccine against tuberculosis (Fine 1995). There are several reports which show that the use of *M. tuberculosis* HSP16.3, increases the efficacy of the BCG vaccination (Shi et al., 2010; Marongiu et al., 2013). Another report in the literature has showed that HSP16.3 and its T-cell epitope synthetic peptide could induce specific antibodies remarkably better than classical tuberculosis vaccine i.e., BCG (Shi et al., 2009). Single or multi-subunit DNA vaccines, over-expressing antigenic proteins from *M. tuberculosis* including HSP16.3 are used to improve the efficacy of BCG in tuberculosis (Shi et al., 2010). Small heat shock protein is also used as carrier protein to develop effective second-generation vaccine (Costa et al., 1998). This approach has been widely used for vaccine development against leprosy, where HSP18 has been used as a carrier protein for the development of second-generation vaccine (Costa et al., 1998). Vaccination is often considered as a safe and effective method to prevent the occurrence of diseases. Altogether, from all the above discussions, it is quite evident that sHSPs have tremendous therapeutic potential (as an agent or a target). This further reinforces the fact that sHSPs are intrinsically involved with the onset of or prevention of several human diseases. Keeping in view the role of ATP and sHSP in the cellular processes of the human body, the role of ATP-sHSP interaction in human diseases is discussed below.

## ROLE OF ATP-SHSP INTERACTION WITH PROLIFERATION OR PREVENTION OF HUMAN DISEASES

### Cardiovascular Disease

*Role of HSP27 and HSP20 in cardiovascular disease*: sHSPs protect cells against ischemia or reperfusion injuries, as evidenced from gene deletion experiments (Sun and MacRae 2005). Over-expressed wild type and non-phosphorylated HSP27 are effective in safeguarding contractile activity and cell integrity, as determined by retention of creatine kinase activity in transgenic mice hearts during ischemia/reperfusion (Hollander et al., 2004). During atrial fibrillation (AF) human body can show response by over-expression of HSP27 to handle the rapid atrial pacing. Mechanism behind this may be the inhibitory effect of angiotensin on atrial remodeling (Wang et al., 2018). HSP27 can also help in prediction of reoccurrence of AF (Marion et al., 2020). A study by Traxler et al. demonstrated that HSP27 can be an independent biomarker for prognosis in chronic heart failure (HF) (Traxler et al., 2017). Wang and others studied the effect of HSP27 on myocardial infarction (MI). They found that deficiency of HSP27 which is specific to cardiomyocytes, can alter the cardiac function negatively like increment in cardiac dysfunction, mortality, and cardiac rupture.

In another example, hearts of double knockout mice that lacked abundant sHSPs like HSP20, showed normal contractility (Morrison et al., 2004). In contrast, hearts of these animals exhibited reduced contractility accompanied by enhanced necrosis and apoptosis when being exposed to ischemia and reperfusion. Thus, HSP20 is essential for optimal recovery from heart attack. Phosphorylation of HSP20 inhibits caspase-3 activation, which arrest apoptosis induced by β-agonist (Morrison et al., 2003). Overall, HSP20 and HSP27 are found to be involved in increased cardiomyocytes contractility, vasorelaxation, smooth muscle relaxation, apoptosis, myocardial contraction, glucose transport, platelet aggregation and ischemia/reperfusion injury (Yu et al., 2019; Zhang et al., 2019; Shan et al., 2021).

### Effect of ATP on HSP27 and HSP20: Implications on Cardiovascular Disease

**FIGURE 1:**
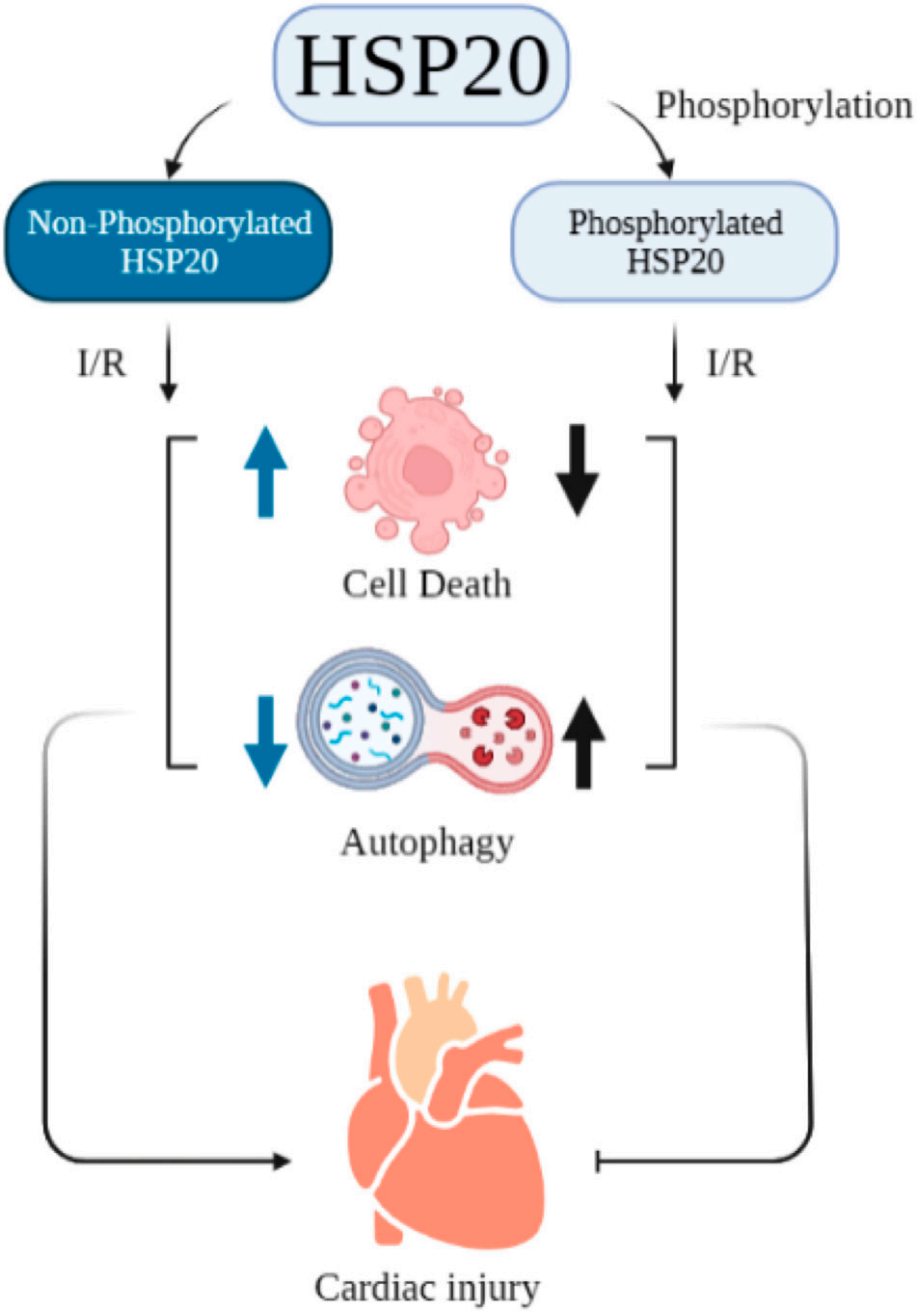
Effect of phosphorylated HSP20 on cardiac injury vs non-phosphorylated one.

The impact of ATP on structure and function of HSP20 and HSP27 are sparsely studied. ATP depletion in endothelial cells resulted in dephosphorylation of HSP27 which caused its translocation into insoluble cellular fraction with altered functional activity towards actin (Loktionova et al., 1996). In contrast, in tubular epithelial cells, ATP depletion caused increased phosphorylation of HSP27 that triggered its migration from cytoskeleton to cytoplasm and promoted actin polymerization (Du et al., 2010). *In vitro* assay using γ-^32^P-ATP revealed that HSP20 is phosphorylated at Ser16 (Sin and Baillie 2015). Ser16 phosphorylation of HSP20 has an impact on cardiac injury. Blocking Ser16 phosphorylation, resulted in increased cell death and reduced autophagy, thereby promoting cardiac injury. A schematic representation of this study is given in Figure 1 (Qian et al., 2009).

Burniston studied the effect of tolerance exercise on the hearts of rats. He claimed that this exercise can help in improving cardiac function and cardiac protection. In this experiment, he also stated that this exercise leads to an increment in phosphorylation of HSP20 at Ser16 (Burniston 2009). Furthermore, the substitution of proline 20 with leucine in HSP20 can diminish the cardio protective activity of its Ser16 (Nicolaou et al., 2008). Guo-Chang Fan and co-workers demonstrated that β-agonist stimulation can lead to phosphorylation of HSP20 which then binds with actin. This binding results in cytoskeleton stabilization and inhibition of apoptosis. Altogether, it can be inferred that ATP possibly controls the phosphorylation of these two sHSPs which influences cardiovascular disease (Figure 1). However, binding affinity of ATP to these two important sHSPs and its effect needs to be assessed carefully.

### Cataract

*Role of αA and αB in cataract*: Mutation and post-translational modifications in sHSP contribute to the cataract formation in mammalian lens (Panda et al., 2015; Hafizi et al., 2021; Khoshaman et al., 2021; Sprague-Piercy et al., 2021). α-crystallin, a major vertebrate eye lens protein is believed to have a chaperone function which plays a major role in maintaining lens transparency, thereby preventing the formation of cataract. Scientists revealed that several post translational modification processes including truncation (Takeuchi et al., 2004), deamidation (Gupta and Srivastava 2004), glycation (Seidler et al., 2004) and phosphorylation (Kamei et al., 2004) decreased the chaperone function of α-crystallin which may be the basis for cataract formation in human lens. The mutations are responsible for autosomal dominant congenital cataract, a common cause of infant blindness, is localized to the arginine 116 (R116) in the αA gene (CRYAA) (Litt et al., 1998). The R116C mutation in αA destroyed its chaperone function (Cobb and Petrash 2000)*.* When a series of transgenic mouse models were created to express R116C mutated αA, it induced lens opacity and structural defects (Hsu et al., 2006). Several other point mutations in αA with autosomal dominant congenital cataracts are: R12C, R21L, R21W, R49C, R54C, G98R and R116H which are well known to impair the structure and function of the protein, thereby inducing cataract formation in human eye lens (Singh et al., 2006; Raju and Abraham 2011). An autosomal recessive congenital cataract causing mutation in αA, W9X, has been also reported in the literature (Pras et al., 2000). Mutations in αB gene have been also reported. Three arginine mutations (R11H, R69C and R120G) were found in the αB gene, which are associated with autosomal dominant congenital cataract in human (Panda et al., 2015). Apart from these arginine mutations, there are other point mutations and truncations in the lens α-crystallin which leads to the formation of the cataract.

### Effect of ATP on α-crystallin’s Chaperone Function

A human lens generally contains 3 mM ATP. Thus, the interaction between α-crystallin and ATP inside lens is highly probable. In fact, ATP binds to β4-β8 groove ACD of αB (Ghosh et al., 2006). The β4-β8 domains are known to interact with C-terminal extension of αB and these domains also interact with stress prone substrate proteins. Thus, ATP binding to αB has altered the chaperone function of the protein. However, ATP hydrolysis is not required for the same (Biswas and Das 2004). ATP enhances the association between chaperone and client proteins, thereby improving the chaperone activity of αA and αB (Muchowski and Clark 1998). ATP binding also improves αB mediated refolding of denatured client proteins like lactate dehydrogenase (Biswas and Das 2004). α-crystallin binds molten globule state of protein like xylanase II that can be refolded in presence of ATP (Nath et al., 2002). The structural stability of α-crystallin is increased in the presence of ATP. In another independent study, it is demonstrated that chaperone-client complexes of α-crystallin remains stable in presence of ATP for 2 weeks under *in vitro* conditions, supporting the notion that ATP promotes association of chaperone-client protein complexes of α-crystallin (Nandi et al., 2020b). In contrast to all these findings, Wang et al. demonstrated that ATP induces conformational changes in α-crystallin that triggers dissociation of chaperone-client protein complexes of α-crystallin (Wang and Spector 2001). This causes release of denatured client protein from α-crystallin, which is then taken up by large heat shock protein to refold. Altogether, it can be inferred that ATP can efficiently regulate chaperone function of α-crystallin and such regulation may be helpful for delaying cataract formation in human. However, the underlying mechanism needs to be further investigated to understand the impact of “α-crystallin-ATP interaction” on the onset of cataract formation.

### Leprosy

*Role of Mycobacterium leprae HSP18 in leprosy*: Among the various antigens over-expressed inside *Mycobacterium leprae*, the etiological pathogen of leprosy, the 18 kDa antigenic protein is an important one. The 18 kDa protein of *M. leprae* is specifically expressed during intracellular growth and may be involved in the survival of *M. leprae* pathogen within macrophages (Dellagostin et al., 1995). Study indicates that the 18 kDa gene may be useful in providing expression signals for foreign gene expression in recombinant BCG vaccines (Dellagostin et al., 1995). Identification of such a gene which is selectively expressed during intracellular growth in macrophages and helps in growth and survival of the pathogen, hinted towards providing a new target for chemotherapy or immunotherapy in the context of the effective treatment of leprosy. Owing to the presence of the “α-crystallin domain” and its sequence identity, this 18 kDa protein is classified as a member of the small heat shock protein family, hence also known as HSP18. Several reports indicate that similar to other well-known sHSPs, HSP18 also exhibits chaperone function by preventing enzymes from thermal inactivation, protecting several thermally and chemically stressed client proteins from aggregation and preventing thermal killing of *E. coli* cells (Lini et al., 2008; Nandi et al., 2013; Nandi et al. 2015a; Nandi et al. 2015b; Nandi et al. 2016; Chakraborty et al., 2018; Nandi et al., 2018; Nandi et al., 2020a; Chakraborty and Biswas 2020; Chakraborty et al., 2021). It has also been found that the over-expression of *M. leprae* HSP18 might facilitate the survival of *M. leprae* under various stressed conditions (Maheshwari and Dharmalingam 2013). In order to find out the molecular basis behind the chaperone function of HSP18, a number of studies have been carried out which includes studies under various thermal and stressed conditions as well as studies in the presence of metal ions and small molecules (Nandi et al., 2015a; Nandi et al., 2015b; Nandi et al., 2016; Chakraborty et al., 2018; Nandi et al., 2018; Nandi et al., 2020a; Chakraborty and Biswas 2020). All these reports indicated that HSP18 is an important leprotic drug target and its chaperoning property is one of the important factors behind controlling the survivability of *M. leprae* pathogen inside the infected hosts.

### Effect of ATP on the Chaperone Function of HSP18

The nutritional requirements and energy metabolism revealed that unlike other obligatory parasitic microorganisms, *M. leprae* does not uptake exogenous ATP from the host species, rather generates its own ATP for energy and other biochemical activities (Lee and Colston 1985; Lee and Colston 1986; Rosa et al., 2021). Aside from energy requirements, ATP is also found to interact with an important antigenic protein HSP18 from *M. leprae* (Nandi et al., 2015a). ATP mostly binds to the β4-β8 strand of HSP18 having binding affinity in sub-micromolar range. In fact, this is the first report which showed that ATP interacts with an antigenic protein of *M. leprae* pathogen. The reversible binding of ATP to *M. leprae* HSP18 enhances its chaperone function without any significant alteration in its conformations. Moreover, ATP is also reported to be involved in the autophosphorylation of HSP18 (Maheshwari and Dharmalingam 2013). Thus, increased chaperone function as a result of HSP18-ATP association along with the autophosphorylation activity in turn may be of significant importance in order to help in the growth and survival of the pathogen *M. leprae* under various physiologically stressed conditions. These findings also indicate that *M. leprae* possesses an ATP binding protein, which evokes the possibility of using ATP competitive antibiotics/inhibitors in the context of effective treatment of leprosy.

### Tuberculosis

*Role of Mycobacterium tuberculosis HSP16.3 in tuberculosis*: Over the years, tuberculosis (TB) remains as one of the major infectious afflictions worldwide, with rising cases of human mortality and morbidity (Preneta et al., 2004; Soong et al., 2018). *Mycobacterium tuberculosis* is the etiological agent of this disease. The characteristic feature of this involved pathogen is that it can remain as a stable dormant bacilli inside the host for years before emerging into active TB (Muchowski et al., 2002). It is possible for the pathogen to remain stable in the hostile environment of host only because of secretion of different immuno-dominant antigens. HSP16.3 is a pivotal one amongst them. This protein was previously known as a 14 kDa antigen, later denoted as HSP16.3 (Panda et al., 2017). It possesses a complex oligomeric assembly of dodecamer (Preneta et al., 2004). HSP16.3 is believed to be overproduced during the latency of *M. tuberculosis* infection and serves as an important diagnostic marker for pleural tuberculosis (Limongi et al., 2011; Zhang et al., 2018; Huang et al., 2021). Garcia *et al.* and Yang et al. have observed that, mycobacteria engulfed by the macrophages, remain in the form of granulomas and produce various mycobacterial products, especially peptides derived from HSP16.3 which act as a vital biomarker for latent tuberculosis and active tuberculosis (Kruh-Garcia et al., 2014; Yang et al., 2018).

HSP16.3 is considered as an important immuno-dominant antigen, which belongs to the family of small heat shock protein and exhibits chaperone activity (Verbon et al., 1992; Chang et al., 1996; Zhang et al., 2018). This protein is highly expressed in the stationary phase of *M. tuberculosis* (Lee et al., 1992). In other words, the molecular chaperone function plays an important role in the growth and survival of *M. tuberculosis* during the latent phase of infection (Yuan et al., 1996). Several attempts have been executed to understand how this immuno-dominant antigen favors the growth and survivability of this pathogen. The studies from Yuan *et al.* have revealed a slower decline in the cell viability in *M. tuberculosis* which are over-expressed with HSP16.3 (Yuan, Crane and Barry third 1996). It also leads to long-term viability during latency and plays an important role in the replication during the initial phase of infection (Yuan et al., 1998). Garbe *et al.* have explored that HSP16.3 plays a prominent role in the survival of this pathogen under nitric oxide stress condition (Garbe et al., 1999). In two independent studies, Timm *et al.* and Hu *et al.* have demonstrated that this antigen is dispensable for the bacterial growth as the multidrug resistant Acr1/HSP16.3 deficient clinical isolate of *M. tuberculosis* do not show impaired replication in macrophages and also exhibit an enhanced rate of growth of the bacilli *in vivo* (Hu et al., 2006; Timm et al., 2006). Also, some studies have emphasized the important role of HSP16.3 in maintaining the dormancy of *M. tuberculosis* during prolonged periods of infection (Hu and Coates 1999, Yuan, Crane and Barry third 1996).

Extensive research is also being conducted to evaluate the potential of this mycobacterial peptide as a successful candidate for developing vaccines. It has been found that the recombinant BCG harboring multistage antigens including HSP16.3 provides long-term protection and increased immune response against the infection caused by *M. tuberculosis* as compared to wild-type BCG vaccine (Shi et al., 2010; Liang et al., 2015). Moreover, Tyagi *et al.* demonstrated that superior booster vaccine can be developed by using these latent antigens such as HSP16.3 which is capable of reducing the risk of developing active tuberculosis by reactivating the latent infection mode (Dey et al., 2011). It has also been observed that the chaperoning ability of HSP16.3 towards the mycobacterial molecules increases the immune response as well as BCG boosting efficacy, which makes it a promising candidate for developing better vaccines for tuberculosis (Taylor et al., 2012). It exhibits this chaperoning activity in an ATP independent manner (Preneta et al., 2004).

### Effect of ATP on the Chaperone Function of HSP16.3

A strong interaction between ATP and HSP16.3 is well established from UV cross-linking experiments and proteolytic studies of HSP16.3 (Muchowski et al., 2002). HSP16.3 has autophosphorylation property *in vitro*, but whether ATP triggers the phosphorylation in HSP16.3 is still unclear. A comparative study revealed the effect of ATP on the recombinant HSP16.3 and human αB to be similar and in both the cases the chaperone activity is significantly increased (Muchowski et al., 2002). In addition to this, from studies of Valdez *et al.*, it is evident that the presence of ATP also prevented the mycobacterial protein from the proteolytic digestion of chymotrypsin (Muchowski et al., 2002). In fact, Dobos and coworkers have identified 122 ATP binding proteins in *M. tuberculosis* and HSP16.3 is one of them (Wolfe et al., 2013). In recent times, ATP competitive inhibitors are being used for the treatment of tuberculosis (Gordon et al., 2015), these inhibitors may affect the “HSP16.3-ATP interaction” which may possibly affect the growth and survival of *M. tuberculosis* in the infected hosts.

## Conclusion

**FIGURE 2:**
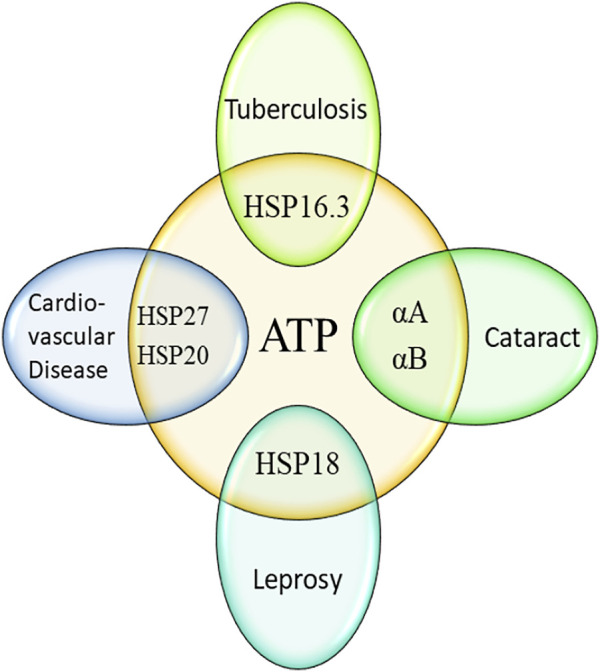
Influence of ATP in human diseases mediated by small heat shock proteins.

This short review clearly depicted that both ATP and different sHSPs play important role in various human diseases (Figure 2). In most cases, the chaperone function of sHSPs is enhanced by the interaction with ATP. The improved chaperone function of many sHSPs in presence of ATP eventually helps in controlling various important diseases. But the improved chaperone function of different mycobacterial sHSPs (HSP18 and HSP16.3) may assist the pathogens (*M. leprae* and *M. tuberculosis*) to survive more in infected hosts. Therefore, these two sHSPs may be a potent target for the development of ATP competitive inhibitors. Interestingly, ATP levels are increased in both leprosy and tuberculosis. Also, the levels of HSP18 and HSP16.3 is elevated in leprosy and tuberculosis, respectively. These sHSPs along with ATP are often used as biomarkers for these two diseases. But, whether the over-expression of these two sHSPs is due to increased levels of ATP is far from clear. Such aspect needs to be explored for the better understanding of host-pathogen interaction.

## Abbreviations

ATP, Adenosine triphosphate; sHSPs, Small heat shock proteins; ACD, α-crystallin domain; αA, αA-crystallin; αB, αB-crystallin; AF, Atrial fibrillation; MI, Myocardial infarction.
